# Supplementary material for: Perioperative and anesthesia-related cardiac arrest and mortality rates in Brazil: A systematic review and proportion meta-analysis
Source: PLoS One. 2020 Nov 2;15(11):e0241751. doi: 10.1371/journal.pone.0241751 (PMC7605701; doi:10.1371/journal.pone.0241751)
Supplement: S2 File — (DOCX) [file pone.0241751.s002.docx]

**S2 File. Search strategy**

**Medline (PubMed)**

(Anesthesia OR Anaesthesia) AND (Anesthesia-Related Cardiac Arrest OR Anaesthesia-Related Cardiac Arrest OR Anesthesia-Related Mortality OR Anaesthesia-Related Mortality OR Heart Arrest OR Arrest, Heart OR Cardiac Arrest OR Arrest, Cardiac OR Asystole OR Asystoles OR Cardiopulmonary Arrest OR Arrest, Cardiopulmonary)

**EMBASE**

((Anesthesia OR Anaesthesia) AND ((Heart Arrest) OR (Arrest, Heart) OR (Anesthesia-Related Cardiac Arrest) OR (Anaesthesia-Related Cardiac Arrest) OR (Anesthesia-Related Mortality) OR (Anaesthesia-Related Mortality) OR (Heart Arrest) OR (Arrest, Heart) OR (Cardiac Arrest) OR (Arrest, Cardiac) OR Asystole OR Asystoles OR (Cardiopulmonary Arrest) OR (Arrest, Cardiopulmonary)))

**LILACS and SciELO**

(Anesthesia OR Anestesia OR Anaesthesia) AND (Heart Arrest OR Paro Cardíaco OR Parada Cardíaca OR Assistolia OR Parada Cardiopulmonar OR Paralisia Cardíaca OR Parada Cardiorrespiratória OR Anesthesia-Related Cardiac Arrest OR Anaesthesia-Related Cardiac Arrest OR Anesthesia-Related Mortality OR Anaesthesia-Related Mortality)
