# Supplementary material for: Perioperative and anesthesia-related cardiac arrest and mortality rates in Brazil: A systematic review and proportion meta-analysis
Source: PLoS One. 2020 Nov 2;15(11):e0241751. doi: 10.1371/journal.pone.0241751 (PMC7605701; doi:10.1371/journal.pone.0241751)
Supplement: S3 File — (DOCX) [file pone.0241751.s003.docx]

**S3 File. Forest Plots**


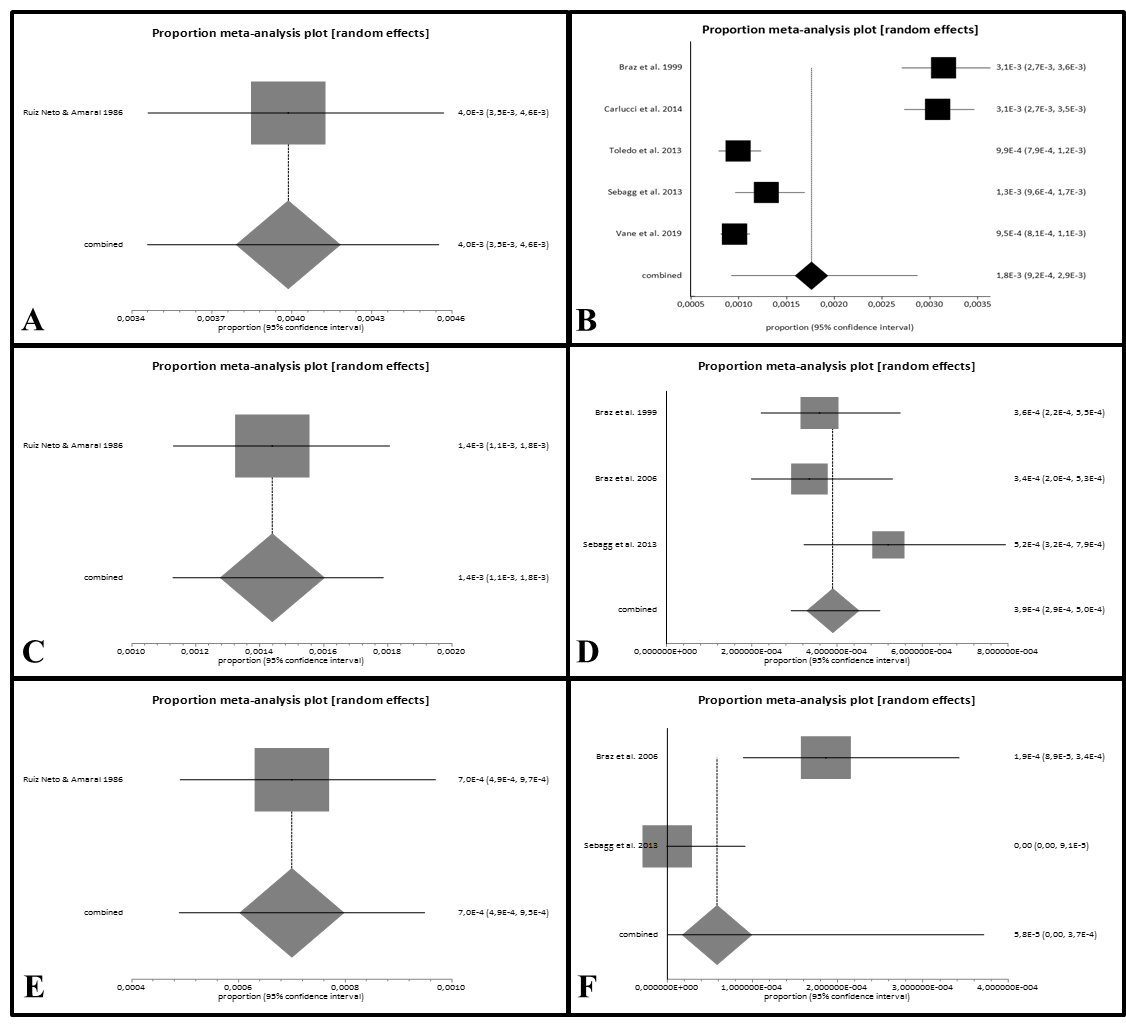


**Fig 1. Pooled analysis of proportions random effects**. Perioperative cardiac arrest - A. Pre-1990: 0.003987 (95% CI: 0.00346-0.00455); B. 1990-2020: 0.001761 (95% CI: 0.000921-0.002868); Anesthesia-related cardiac arrest - C. Pre-1990: 0.001439 (95% CI: 0.001129-0.001786); D. 1990-2020: 0.00039 (95% CI: 0.000293-0.000501); Entirely anesthesia-related cardiac arrest - E. Pre-1990: 0.0007 (95% CI: 0.000489-0.000949); F. 1990-2020: 0.000058 (95% CI: 0-0.000372).


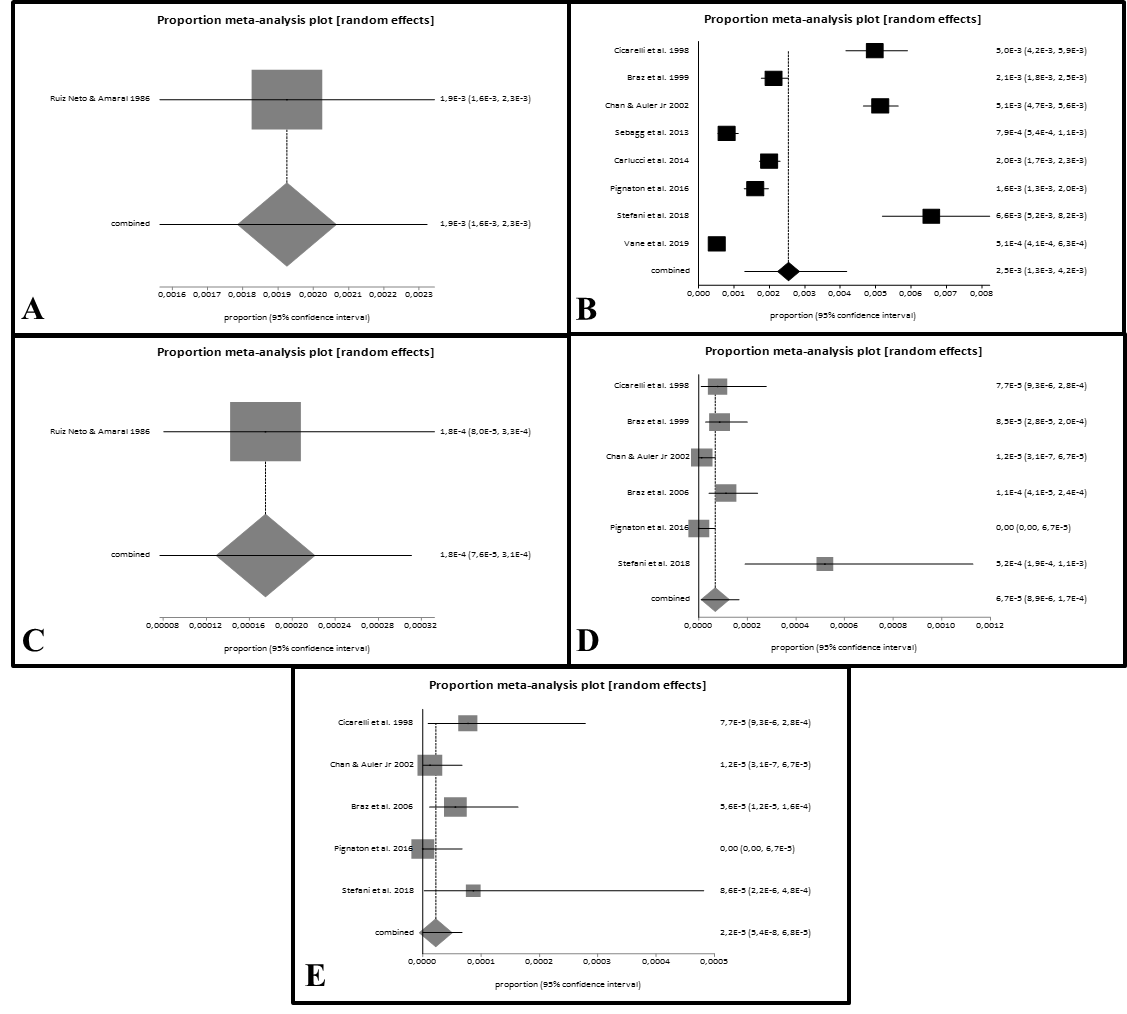


**Fig 2. Pooled analysis of proportions random effects**. Perioperative mortality - A. Pre-1990: 0.001925 (95% CI: 0.001564-0.002324); B. 1990-2020: 0.002540 (95% CI: 0.001301-0.004186); Anesthesia-related mortality - C. Pre-1990: 0.000175 (95% CI: 0.000076-0.000311); D. 1990-2020: 0.000067 (95% CI: 0.000009-0.000166); Entirely anesthesia-related mortality - E. 1990-2020: 0.000022 (95% CI: 0.000005-0.000068).
