# Supplementary material for: Perioperative and anesthesia-related cardiac arrest and mortality rates in Brazil: A systematic review and proportion meta-analysis
Source: PLoS One. 2020 Nov 2;15(11):e0241751. doi: 10.1371/journal.pone.0241751 (PMC7605701; doi:10.1371/journal.pone.0241751)
Supplement: S1 Table — (DOCX) [file pone.0241751.s004.docx]

**S1 Table. Standard form used to extract information from the studies**

| Investigator(s) and year of publication |
| --- |
| Data source and recruitment year(s) |
| Median year of recruitment |
| Patients (n) |
| Perioperative CA (n) |
| Anesthesia-related CA (n) |
| Entirely anesthesia-related CA (n) |
| Perioperative mortality (n) |
| Anesthesia-related mortality (n) |
| Entirely anesthesia-related mortality (n) |
| Excluded patients |

*Abbreviations.* CA: cardiac arrest
