# Supplementary material for: Perioperative and anesthesia-related cardiac arrest and mortality rates in Brazil: A systematic review and proportion meta-analysis
Source: PLoS One. 2020 Nov 2;15(11):e0241751. doi: 10.1371/journal.pone.0241751 (PMC7605701; doi:10.1371/journal.pone.0241751)
Supplement: S2 Table — (DOCX) [file pone.0241751.s005.docx]

**S2 Table. Critical appraisal results for included studies using the JBI Prevalence Critical Appraisal Checklist**

|  | Target population | Sampling | Sample size | Description of participants and setting | Coverage of identify sample | Methods to identify outcome | Reliability in outcome measurement | Appropriate statistical analysis | Response rate | **Total** |
| --- | --- | --- | --- | --- | --- | --- | --- | --- | --- | --- |
| Braz et al., 1999 | Y | Y | Y | Y | Y | Y | Y | N | Y | 8/9 |
| Braz et al., 2006 | Y | Y | Y | Y | Y | Y | Y | Y | Y | 9/9 |
| Carlucci et al., 2014 | U | Y | Y | Y | N | Y | Y | Y | N | 6/9 |
| Chan & Auler Jr, 2002 | Y | Y | Y | N | Y | Y | Y | N | Y | 7/9 |
| Cicarelli et al., 1998 | Y | Y | Y | N | Y | Y | Y | N | Y | 7/9 |
| Pignaton et al., 2016 | Y | Y | Y | Y | Y | Y | Y | Y | Y | 9/9 |
| Toledo et al., 2013 | U | Y | Y | Y | Y | Y | Y | Y | Y | 8/9 |
| Sebbag et al., 2013 | Y | Y | Y | Y | Y | Y | Y | Y | Y | 9/9 |
| Stefani et al., 2018 | Y | Y | Y | Y | Y | Y | Y | Y | Y | 9/9 |
| Ruiz Neto & Amaral, 1986 | Y | Y | Y | Y | Y | Y | Y | N | Y | 8/9 |
| Vane et al., 2019 | Y | Y | Y | Y | Y | Y | Y | Y | Y | 9/9 |

Y: Yes; N: No; U: Unclear
