# Supplementary material for: Perioperative and anesthesia-related cardiac arrest and mortality rates in Brazil: A systematic review and proportion meta-analysis
Source: PLoS One. 2020 Nov 2;15(11):e0241751. doi: 10.1371/journal.pone.0241751 (PMC7605701; doi:10.1371/journal.pone.0241751)
Supplement: Attachment — Submitted filename: Response to Reviewers.docx [file pone.0241751.s006.docx]

**Answers to the Editor**

**Answer:** First, we would like to thank the Editor for taking the time to review our manuscript and make important comments and suggestions. We tried our best to answer the questions and make the required changes in the manuscript after considering the suggestions, which certainly improved the revised manuscript.

1. “Please ensure that your manuscript meets PLOS ONE's style requirements, including those for file naming. The PLOS ONE style templates can be found at

<https://journals.plos.org/plosone/s/file?id=wjVg/PLOSOne_formatting_sample_main_body.pdf> and <https://journals.plos.org/plosone/s/file?id=ba62/PLOSOne_formatting_sample_title_authors_affiliations.pdf>”

**Answer:** We apologize. Our revised manuscript meets PLOS ONE´s style requirements.

2. “In the Methods, please specify any assessment of risk of bias that may affect the cumulative evidence (e.g., publication bias, selective reporting within studies). Please ensure that the specific method of assessment (funnel plot, Egger's test, Begg's test, etc) is mentioned.”

**Answer:** We agree with the suggestion, but we would like to clarify that our review is limited by a paucity of studies. Thus, unfortunately, we were not able to analyze publication bias because there were fewer than ten eligible studies addressing each outcome in the meta-analysis; otherwise, the findings from the assessment of risk of bias would not be reliable. Despite the heterogeneity and small number of studies, the meta-analyses showed consistent results that strongly reinforce the relevance of our systematic review. Thus, we included the information as one of the limitations of our review in the discussion section.

3. “In the Methods, please specify how study quality was assessed.”

**Answer:** We agree with the comment. We have added the topic “Assessment of methodological quality” to the methods, specifying the assessment of study quality using the Joanna Briggs Institute (JBI) - Critical Appraisal Tool for Prevalence Studies.

4. “Thank you for stating the following in the Acknowledgments Section of your manuscript:

R.S., M.P. and J.B.C. received a scholarship from The National Council for
Scientific and Technological Development (CNPq) and L.G.B. received a fellowship
from CNPq (304466/2015-8). M.P.M. received a scholarship from Coordination of
Improvement for Higher Academic Staff (CAPES). CNPq and CAPES are Brazilian
governmental agency dedicated for promoting scientific research.

We note that you have provided funding information that is not currently declared in your Funding Statement. However, funding information should not appear in the Acknowledgments section or other areas of your manuscript. We will only publish funding information present in the Funding Statement section of the online submission form. Please remove any funding-related text from the manuscript and let us know how you would like to update your Funding Statement. Currently, your Funding Statement reads as follows: The authors received no specific funding for this work.”

**Answer:** We apologize, and we have corrected the funding statement.

5. “Please include captions for your Supporting Information files at the end of your manuscript, and update any in-text citations to match accordingly. Please see our Supporting Information guidelines for more information: http://journals.plos.org/plosone/s/supporting-information.”

**Answer:** The captions for the supporting information files are at the end of our revised manuscript.

**Answers to the Reviewers**

**Reviewer #1**

“Thank you for the opportunity to review this manuscript.”

**Answer:** We would like to thank you for taking the time to review our manuscript and to make important suggestions. We tried our best to answer the questions and make the required changes in the manuscript after considering the suggestions, which certainly improved the revised manuscript.

1. “I have a few comments and questions for the authors. Under methods; you've indicated you adhered to the MOOSE guidelines, but this is not a methodology, this is a guideline that serves as a checklist for reporting observational studies in systematic reviews and meta-analyses. What methodological framework, or handbook did you use to conduct this systematic review and meta-analysis? The Cochrane Handbook? JBI? CRD York? You must always state the methods you followed to conduct this study, in addition to the relevant reporting guideline, which you must use to report your study, in this case it would be PRISMA. My recommendation would be that authors indicate the methods they used to conduct the study, and include the PRISMA checklist indicating where each of the items in the guideline was reported. Some of the diagrams are confusing, primarily due to labelling, e.g. the references workflow diagram is your PRISMA Diagram, but it's not labelled adequatey.”

**Answer:** As suggested by the reviewer, we described in the first paragraph of the methods section that The Cochrane Handbook for Intervention Reviews guided our choice of methods. Our reporting adheres to the Preferred Reporting Items for Systematic Reviews and Meta-analyses (PRISMA) statement. We followed the PRISMA diagram, as requested.

2. “The manuscript could also benefit from copyediting, there are instances where the syntax is unclear.”

**Answer:** We apologize. The original version of the manuscript was copyedited by a native English speaker. The revised manuscript was copyedited again and improved.

**Reviewer #2**

1. “This paper is a review and meta-analysis whose goal is to summarize the evidence surrounding changes in perioperative and anesthesia-related cardiac arrest and mortality rates in Brazil.”

**Answer:** We would like to thank you for taking the time to review our manuscript and to make important comments. We tried our best to answer the comments and to clarify the revised manuscript.

2) “I am not an expert on cardiac arrest and/or surgery in Brazil, so I can only comment on the statistical part of this paper. The writing is clear and I think it is possible from this text to evaluate what the statistical analysis that the authors have performed.
My main concern is that the meta-analysis reveals quite large amounts of heterogeneity for several important quantities; Tables 3 and 4 suggest that I2 can be as high as 99 percent, and is higher than 80 percent for 4 out of 6 of the quantities. This is somewhat subjective, but I am not confident that a meta-analysis can be meaningfully performed if there is this much heterogeneity in the effects being aggregated. The authors might at least consider reporting additional statistics intended to help the reader understand how much heterogeneity there is in the estimated true effect sizes.”

**Answer:** We agree with the comment that most of the proportion meta-analyses are heterogeneous. As previously described in the original manuscript (limitation paragraph of the discussion section), a random-effects model with inverse Freeman-Tukey double arcsine transformation (Freeman-Tukey transformation) was applied to minimize this heterogeneity when assessing the trends between the two time periods. If we had used a fixed-effects model (please see the table below), the results of the event rates and the statistical analyses (P values) would be different and incorrect. Thus, we were aware of the high degree of heterogeneity, and we applied the best options to minimize it following Higgins JP al.’s (2003) instructions - “Measuring inconsistency in meta-analyses”.

| **Time period** | **Studies**  **n** | ***I*^2^**  **%** | **Events**  **n** | **Patients**  **n** | **Proportion meta-analysis**  **per 10,000 anesthetics**  **(95% CI)** | **P value for subgroup** |
| --- | --- | --- | --- | --- | --- | --- |
|  |  |  |  |  |  | Pre-1990 *versus* 1990-2020 |
| Anesthesia-related mortality - **Random-effects model (included in the manuscript)** | | | | | |  |
| **Pre-1990** | 1 | NA | 9 | 51,422 | 1.75 (0.76-3.11) | 0.5404 |
| **1990-2020** | 6 | 81.0 | 20 | 287,402 | 0.67 (0.09-1.66) |  |
|  |  |  |  |  |  |  |
| Anesthesia-related mortality - **Fixed-effects model (incorrect presentation)** | | | | | |  |
| **Pre-1990** | 1 | NA | 9 | 51,422 | 1.75 (0.76-3.11) | ***< 0.05*** |
| **1990-2020** | 6 | 81.0 | 20 | 287,402 | ***0.41 (0.18-0.71)*** |  |

**Table. Proportion meta-analysis of mortality rates in Brazilian studies by time period**

3) “Minor comments: * pg 5 - I believe this should read 'sample size' instead of 'simple size'”

**Answer:** We apologize for our mistake. We corrected the word to “sample”.
