# Supplementary material for: Perioperative and anesthesia-related cardiac arrest and mortality rates in Brazil: A systematic review and proportion meta-analysis
Source: PLoS One. 2020 Nov 2;15(11):e0241751. doi: 10.1371/journal.pone.0241751 (PMC7605701; doi:10.1371/journal.pone.0241751)
Supplement: Attachment — Submitted filename: Response to Reviewers.docx [file pone.0241751.s007.docx]

**Answers to the Reviewer #3**

“Interesting paper with only some issues, mainly related to abstract.

Abstract:

methods>proportion meta-analysis is not clear

methods>4 outcomes as primary outcome are too many. Authors should decide one primary outcome and other as co end points.

methods>time periods are not defined anywhere.”

**Answer:** We would like to thank you for taking the time to review our manuscript and the compliments on our study. We appreciated the suggestions and we incorporated them into the abstract, which we believe is now improved.
